# New 2D Metal‐Organic Monoacid Framework (MOmAF): Realization of Extreme Water Repellence

**DOI:** 10.1002/smll.202404224

**Published:** 2024-07-31

**Authors:** Ting Chen, Xiuming Wei, Thomas Fabiani, Baoyu Liu, Yaohao Yang, Allana Lewis, Nurul A. Mazlan, Fraz Saeed Butt, Siyu Chen, Qinfeng Gu, Norbert Radacsi, Huanting Wang, Maria Grazia De Angelis, Shuiqing Yang, Haiqun Chen, Yi Huang

**Affiliations:** ^1^ School of Engineering Institute for Materials & Processes The University of Edinburgh Robert Stevenson Road Edinburgh EH9 3FB UK; ^2^ School of Chemical Engineering and Light Industry Guangdong Provincial Key Laboratory of Plant Resources Biorefinery Guangzhou Key Laboratory of Clean Transportation Energy Chemistry Guangdong University of Technology Guangzhou 510006 China; ^3^ Jieyang Branch of Chemistry and Chemical Engineering Guangdong Laboratory (Rongjiang Laboratory) Jieyang 515200 China; ^4^ Australian Synchrotron Clayton, Melbourne Victoria 3168 Australia; ^5^ Department of Chemical and Biological Engineering Monash University Clayton Victoria 3800 Australia; ^6^ Jiangsu Dingying New Materials Co., Ltd. Changzhou Jiangsu 213031 China; ^7^ Key Laboratory of Advanced Catalytic Materials and Technology Advanced Catalysis and Green Manufacturing Collaborative Innovation Center Changzhou University Changzhou Jiangsu Province 213164 China

**Keywords:** interfacial synthesis, metal‐organic monoacid framework (MOmAF), superhydrophobic, water stability, zinc‐organic monoacid framework (ZOmAF)

## Abstract

Metal‐organic frameworks (MOFs) are normally moisture‐sensitive and unstable in aqueous environments, which has considerably limited their practical applications because water/moisture is ubiquitous in many industrial processes. New materials with superior water stability are, therefore, in great demand and vital to their practical applications. Here, a novel oil/water interfacial assembly strategy is demonstrated for the synthesis of a new class of metal‐organic monoacid framework (MOmAF) with exceptional water stability and chemical stability. Superhydrophobic 2D sheets are synthesized at room temperature, while 1D nanotubes are obtained via the self‐scrolling of their 2D sheets for the first time. In addition, a simple sequential drop‐casting method is developed to coat as‐synthesized MOmAF structures onto porous membranes. This can potentially open up new avenues in the design of superhydrophobic self‐cleaning MOmAF materials without tedious post‐synthetic modifications and usher in a new class of materials meeting industrial needs.

## Graphical abstract

An oil/water interfacial synthesis method is designed to prepare novel 1D and 2D metal‐organic monoacid framework (MOmAF) sheets. Importantly, all the synthesized materials are intrinsically hydrophobic and show extraordinary stability under aqueous conditions. This new synthesis concept can be applied to design further examples of water‐stable MOmAFs for various applications, such as gas adsorption, catalysis, and membrane separation.

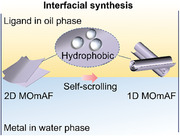

## 1 Introduction

Metal‐organic frameworks (MOFs) are an evolving class of highly porous, crystalline materials composed of metal ions bridged by organic linkers.^[^ ^1^, ^2^ ^]^ MOFs have received enormous attention over the last decades because of their unique chemical versatility and tremendous structural flexibility. MOF‐based materials have thus been extensively studied for a wide range of applications, such as electrochemical sensing,^[^ ^3^ ^]^ catalysis,^[^ ^4^ ^]^ energy storage,^[^ ^5^ ^]^ gas adsorption/separation,^[^ ^6^ ^]^ and liquid separation.^[^ ^7^ ^]^ In particular, 2D MOF nanosheets^[^ ^8^ ^]^ and 1D MOF nanotubes,^[^ ^9^ ^]^ nanorods,^[^ ^10^ ^]^ and nanowires^[^ ^10^, ^11^ ^]^ have drawn intensive research interest in recent years because they represent a novel and emerging research area where traditional materials exhibit exceptional low‐dimensional properties, such as a high aspect ratio and plentiful active sites,^[^ ^8^, ^9^, ^10^, ^11^ ^]^ which are not commonly found in natural materials. In addition to these advantageous properties, it is highly desirable to put significant effort into their large‐scale production and real‐world applications.

2D MOF nanosheets have been regarded as the most recent form factor for MOF‐based materials. Their layered structure and ultrathin thickness offer a novel combination of properties, such as highly accessible active sites and optimized mass/charge transfer kinetics.^[^ ^8^, ^12^ ^]^ These extraordinary properties of 2D materials have led to a significant enhancement of their performance in energy‐related areas, including sensing,^[^ ^13^ ^]^ catalysis,^[^ ^14^ ^]^ and separation.^[^ ^15^ ^]^ Moreover, the intrinsic structural flexibility of 2D MOF nanosheets demonstrates their great potential as building blocks for the assembly of novel 1D MOF nanostructures. The assembled 1D MOF nanostructures could not only inherit the fascinating properties from the hosting 2D MOF nanosheets but also generate unique physicochemical properties from the 1D structure.^[^ ^10^, ^16^, ^17^ ^]^ However, only very few 1D MOF nanostructures have been reported so far in comparison to the rapid development of 2D MOFs, owing to the difficulty in directing the linear assembly of MOF crystals. The existing 1D MOF nanostructures have been synthesized either through a template approach^[^ ^18^ ^]^ or a non‐template approach.^[^ ^19^ ^]^ In the template approach, rationally designed polymers,^[^ ^20^ ^]^ 1D nanomaterials (e.g., ZnO nanorods,^[^ ^21^ ^]^ Te nanowires,^[^ ^22^ ^]^ and 1D GO nanoscrolls^[^ ^11^ ^]^), and membrane channels^[^ ^10^ ^]^ have been employed as templates to direct the growth of 1D MOFs. For example, Arbulu et al.^[^ ^10^ ^]^ synthesized 1D ZIF‐8 nanowires, nanorods, and nanotubes using track‐etched polycarbonate (PCTE) membrane channels as the template. Despite the effectiveness of template approaches to achieve 1D MOFs with well‐regulated structures, there are still concerns about the tedious preparation procedures when coming into industrial‐scale production. In contrast, the template‐free approach, i.e., direct synthesis methods, remarkably simplified the preparation procedure for 1D MOFs.^[^ ^23^ ^]^ Recently, 1D MOF structures have been synthesized directly via the 2D MOF “rolling‐up” method,^[^ ^24^ ^]^ crystal phase transformation,^[^ ^25^ ^]^ controlled metal ion assembly, and guided growth with rationally designed organic ligands.^[^ ^26^ ^]^ Although great progress has been made in recent years, the direct syntheses of 1D MOFs are still at an early developmental stage. Therefore, facile and controllable direct synthesis approaches for 1D MOFs are still highly desired, especially methods that ensure energy efficiency and the ability to scale on demand.

Apart from an effective synthesis approach to the preparation, exceptional stability of the synthesized MOF material is equally important for promoting their practical applications in various fields.^[^ ^27^ ^]^ Although there is an increasing trend in the discovery of MOFs that exhibit remarkable chemical stability in recent years, many MOFs are known to be sensitive to water content.^[^ ^27^ ^]^ The instability issue has considerably limited any real‐world application of MOFs since water or moisture is ubiquitous in most industrial processes. To overcome this problem, researchers have made great efforts to enhance the water stability of MOFs. Existing methods to enhance the water stability of MOFs can be categorized into two types: (1) Synthesizing MOFs with strong coordination bonds by employing high oxidation state metals (such as Fe^3+^, Al^3+^, and Zr^4+^) and carboxylic acid ligands,^[^ ^28^ ^]^ or using alkali metals and nitrogen‐donor ligands.^[^ ^29^ ^]^ (2) Functionalizing existing MOFs by the hydrophobic functionalization of organic linkers,^[^ ^30^ ^]^ or the hydrophobic post‐modification of external MOF surfaces.^[^ ^31^ ^]^ For example, Omary et al.^[^ ^31^ ^]^ demonstrated that highly hydrophobic fluorous MOFs exhibit remarkable vapor and water stability. Despite that, direct synthesis of water‐stable MOFs is still very challenging. However, the synthesis of new water‐stable MOF analogous materials sharing similar interests and markets may be an alternative.

**Scheme 1:**
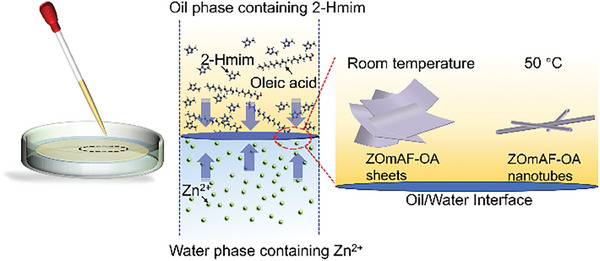
Schematic illustration of the oil/water interfacial assembly process for ZOmAF‐OA structures.

In this paper, we report an oil/water interfacial assembly strategy for the preparation of exceptionally water‐stable self‐scrolling metal‐organic monoacid frameworks (MOmAF) initially using a modified zeolitic imidazolate framework (ZIF) synthesis recipe (see 2. Methods in Supporting Information). 2D self‐scrolling zinc‐organic monoacid framework (ZOmAF) sheets were produced at the oil/water interface at room temperature through the coordination of zinc ions from the water phase and imidazole ligands from the oil phase. Interestingly, when increasing the temperature to 50 °C, 2D ZOmAFs were observed to self‐scroll, producing 1D ZOmAF nanoscrolls and nanotubes (**Scheme** **1**). These synthesized ZOmAFs showed highly hydrophobic characteristics with water contact angles up to ≈150° ± 2°. This excellent hydrophobicity was attributed to the decoration of oleic acid (OA) molecules on the ZOmAF structures during synthesis. OA is an amphiphilic fatty acid that naturally exists in various animal fats and vegetable oils. For the first time, we report the direct synthesis of superhydrophobic ZOmAF sheets and their 1D nanotubes without involving any fluorochemicals. More importantly, the as‐synthesized ZOmAFs (denoted as ZOmAF‐OAs) demonstrated superior water stability and chemical stability, which are significant for their practical applications. Furthermore, a simple sequential drop‐casting method was developed to coat ZOmAF‐OA structures on porous membranes for molecular separations.

## 2 Results and Discussion

ZOmAF‐OA structures were prepared by a simple oil/water interfacial assembly process. An aqueous solution of Zn(NO_3_)_2_.6H_2_O (1 M, 5 mL) was first added to a clean petri dish, and then an OA solution of 2‐methylimidazole (2‐Hmim, 0.4 M, 1 mL) was added cautiously onto the surface of the aqueous solution to form an oil/water interface. At the formed interface, zinc ions (Zn^2+^) from the aqueous phase coordinate with imidazole linkers from the oil phase to produce ZIF structures. OA molecules with long aliphatic chains are used to decorate ZIF structures to form a shell layer with superior hydrophobicity. The synthesized product was thus named ZOmAF‐OA. The two‐phase solution was aged for 24 h at room temperature and 50 °C to produce 2D ZOmAF‐OA sheets and 1D ZOmAF‐OA nanotubes, respectively.

**Figure 1:**
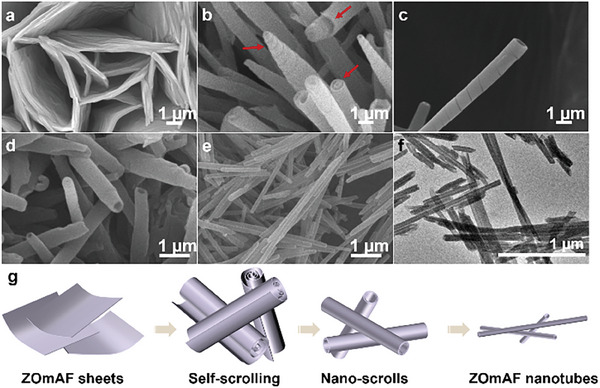
Morphologies of the synthesized ZOmAF‐OAs. SEM images of a) 2D ZOmAF‐OA sheets synthesized by interfacial assembly at room temperature; b) self‐scrolled 2D ZOmAF‐OA with Archimedean spiral morphology formed at 50 °C for 12 h; c) self‐scrolled ZOmAF‐OA with rolled‐up straw‐like morphology formed at 50 °C for 12 h; d) 1D ZOmAF‐OA nanoscrolls synthesized at 50 °C for 16 h; e) 1D ZOmAF‐OA nanotubes synthesized at 50 °C for 48 h; f) TEM image of 1D ZOmAF‐OA nanotubes; g) schematic illustration showing the self‐scrolling process of 2D ZOmAF‐OA layers to form 1D ZOmAF‐OA nanotubes.

Morphologies of synthesized ZOmAF‐OA structures are observed using a scanning electron microscope (SEM) (**Figure** **1**a–e). As shown in Figure 1a, ZOmAF‐OAs synthesized at room temperature showed 2D layered morphologies. The width of these 2D ZOmAF‐OA sheets was estimated to vary from 2 µm to 10 µm, with an average thickness of ≈400 nm. Additional SEM images of the 2D ZOmAF‐OA layers can be found in Figure S1 (Supporting Information).

Interestingly, unlike traditional 2D MOFs, which usually have rigid structures (such as ZIF‐L), these 2D ZOmAF‐OA layers appeared to be soft and flexible. When increasing the synthesis temperature to 50 °C, large flexible 2D ZOmAF‐OA layers tend to self‐scroll, forming an Archimedean spiral morphology (Figure 1b) or a rolled‐up straw‐like morphology (Figure 1c). Additional SEM images of the scrolling ZOmAF‐OAs can be found in Figure S2 (Supporting Information). Moreover, an increase in the synthesis time promoted the scrolling of the majority of ZOmAF‐OA layers, including those with small lateral size, forming ZOmAF‐OA nanoscrolls with a mean diameter of ≈357 nm and length of ≈2.0 µm (Figure 1d). A further increase in the synthesis time favors the 1D growth of ZOmAF‐OA scrolls, producing 1D ZOmMAF‐OA nanotubes with a uniform morphology. As shown in Figure 1e, ZOmAF‐OA nanotubes with a mean diameter of ≈105 nm and length of ≈3.2 µm were obtained when the synthesis time was increased to 48 h. The size distribution of the nanotube diameter and length can be found in Figure S3 (Supporting Information). The transmission electron microscopy (TEM) image in Figure 1f confirms the hollow structure of the synthesized ZOmAF‐OA nanotubes. Based on SEM and TEM characterizations, a schematic illustration showing the formation of 1D ZOmAF‐OA nanotubes by the self‐scrolling of 2D ZOmAF‐OA layers is presented in Figure 1g. For the first time, we achieved the direct synthesis of 1D ZOmAF nanotubes through the self‐scrolling of 2D ZOmAF sheets. Compared to previously reported template methods for 1D ZOmAFs,^[^ ^17^, ^20^, ^21^, ^22^ ^]^ our approach has a remarkably simplified preparation procedure. Therefore, the new interfacial synthesis method developed in this work is of great significance for promoting the preparation of 1D ZOmAFs.

To scrutinize the chemical and structural properties of the interfacially synthesized ZOmAF‐OAs, FTIR spectra of 2D ZOmAF‐OA sheets and 1D ZOmAF‐OA nanotubes were analyzed (Figure S4, Supporting Information). Meanwhile, FTIR spectra of ZIF‐L and ZIF‐8, which are two of the most extensively studied ZIFs prepared from the same metal and ligand source, were also studied as controls (Figure S4, Supporting Information). The FTIR spectrum of ZIF‐L showed characteristic peaks at 1422 cm^−1^ and 1306 cm^−1^, corresponding to imidazole ring stretching and C‐N bonds in the imidazole group, respectively. The FTIR spectrum of ZIF‐8 showed an intense peak at 1336 cm^−1^, which was ascribed to the in‐plane bending vibration of the imidazole ring. In comparison, FTIR spectra of both the ZOmAF‐OA sheet and ZOmAF‐OA nanotube showed new intense peaks at 2920 cm^−1^ and 2850 cm^−1^ (Figure S4, Supporting Information), which were attributed to the C‐H stretching vibration from the long aliphatic chain of OA. This confirms the existence of OA molecules in the interfacially synthesized ZOmAF‐OA structures.

**Figure 2:**
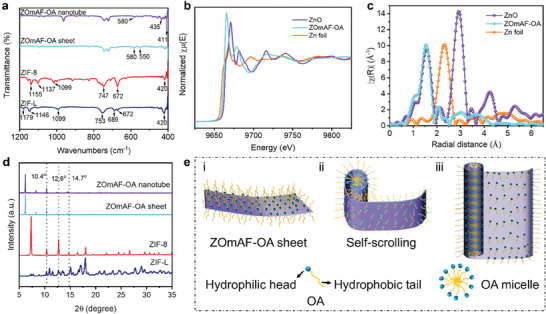
Structural characterizations and the formation mechanism of ZOmAF‐OAs. a) FTIR spectra of ZIF‐L, ZIF‐8, and the synthesized ZOmAF‐OA sheet as well as the ZOmAF‐OA nanotube; b) XANES spectra and c) EXAFS spectra of ZOmAF‐OA sheet and Zn foil; d) X‐ray diffraction (XRD) patterns of ZIF‐L, ZIF‐8, ZOmAF‐OA sheet, and ZOmAF‐OA nanotube; e) schematic diagram showing the proposed formation mechanism of ZOmAF‐OA nanotubes from ZOmAF‐OA sheet.

**Figure** **2**a presents the FTIR spectra of different ZIF structures in the lower wavenumber region. For ZIF‐L, peaks at 1179 cm^−1^, 1146 cm^−1^, and 1099 cm^−1^ correspond to the out‐of‐plane bending of the imidazole ring. The peak at 753 cm^−1^ is assigned to the wagging vibration of N‐H bonds, and peaks at 689 cm^−1^, 672 cm^−1^, and 420 cm^−1^ are attributed to Zn‐N bonds. For ZIF‐8, peaks at 1155 cm^−1^, 1137 cm^−1^, and 1099 cm^−1^ correspond to the out‐of‐plane bending of the imidazole ring. The peak at 747 cm^−1^ is assigned to the wagging vibration of N‐H bonds, and peaks at 672 cm^−1^ and 420 cm^−1^ are attributed to Zn‐N bonds. For the ZOmAF‐OA sheet, weak peaks at 580 cm^−1^ and 550 cm^−1^ are assigned to Zn‐O bonds. For the ZOmAF‐OA nanotube, weak peaks in the range of 435 cm^−1^ to 580 cm^−1^ and the peak at 411 cm^−1^ can be assigned to Zn‐O bonds. The FTIR analysis results suggest that OA molecules arlinked with zinc centers via Zn‐O bonds. A summary of the characteristic peaks of all the tested ZIFs can be found in Tables S1–S4 (Supporting Information). Figure 2b presents the Zn K‐edge X‐ray absorption near‐edge structure (XANES) spectra of the synthesized ZOmAF‐OA sheet. The XANES spectra show a decrease in the absorption threshold energy of Zn ions in ZOmAF‐OA compared to that in the ZnO standard. The diminished absorption threshold energy for Zn ions in ZOmAF‐OA indicates a comparatively reduced oxidation state for Zn ions in ZOmAF‐OA relative to ZnO. Figure 2c shows the extended X‐ray absorption fine structure (EXAFS) spectra of the ZOmAF‐OA sheet and Zn metal foil.^[^ ^32^, ^33^ ^]^ Zn metal foil, ZnO, and ZOmAF‐OA exhibit significant differences in the EXAFS spectra, suggesting different Zn^2+^ coordination environments.^[^ ^34^, ^35^ ^]^ The absence of a Zn‐Zn peak in the EXAFS spectra indicates that Zn^2+^ ions are atomically dispersed within the ZOmAF‐OA framework, rather than forming Zn‐Zn bonds as seen in bulk zinc structures. This suggests that the Zn^2+^ ions are well‐coordinated within the ZOmAF‐OA framework. Additionally, atomistic simulation was performed to further elucidate the equilibrium configuration of the structure of the synthesized ZOmAF‐OA. Each slab of ZOmAF‐OA was built considering zinc atoms coordinating oxygens from the OAs in the tetrahedral site, as suggested by EXAFS results. More details on the simulation methodology are reported in Supporting Information in S1.2.4. As suggested by the simulated structure presented in Figure S5 (Supporting Information), the synthesized ZOmAF‐OA shows a 2D structure in which zinc atoms lie on the basal plane. Fatty acids are in both upward and downward orientations with respect to the plane itself, forming two layers of organic tails, tilting and packing with an ordered structure, at 298 K. Layers of ZOmAF‐OA proved to stack one over each other, with an equilibrium distance (between Zn atoms of different layers) equal to 40.85 Å (−3.77% of experimental d‐space). The structure obtained by molecular simulation indicated a formula of Zn(O_2_C_18_H_33_)_2_ that agrees well with elemental analysis results (see the text accompanying Figure S5, Supporting Information).

The crystal structures of ZOmAF‐OA sheets and nanotubes were determined by X‐ray diffraction (XRD) and compared with ZIF‐L and ZIF‐8, as shown in Figure 2d. The XRD pattern of ZIF‐L exhibited characteristic peaks that were consistent with previously reported peaks. The XRD pattern of ZIF‐8 showed characteristic peaks at 2θ = 7.3°, 10.4°, 12.7°, 14.7° 16.4°, and 18.1°, which were identical to those reported in previous studies. XRD patterns of ZOmAF‐OA sheets and ZOmAF‐OA nanotubes both showed weak peaks at 2*θ* = 10.4°, 12.6°, and 14.7°, which were consistent with peaks in the XRD pattern of ZIF‐8 and correspond to the crystallographic planes of (200), (211), and (220). In addition, ZOmAF‐OA sheets and nanotubes presented two new peaks at 2*θ* = 6.3° and 8.4°, suggesting that their crystal structures were different from those of either ZIF‐L or ZIF‐8. Based on the above structural analysis of ZOmAF‐OAs, a formation mechanism of the 1D ZOmAF‐OA structure from 2D ZOmAF‐OA sheets was proposed and depicted in Figure 2e. In brief, zinc ions from the aqueous phase and imidazole ligands from the oil phase coordinated at the 2D confined interface to form 2D ZOmAF layers. Meanwhile, the amphiphilic OA molecule, which is composed of a hydrophilic carboxylic acid head and a hydrophobic carbon tail, is regularly adsorbed at the interface with the hydrophilic head facing the water phase and the hydrophobic tail facing the oil phase. Therefore, during the formation of the ZOmAF structure, OA not only acted as the solvent for imidazole ligands in the oil phase but also partially bonded with zinc ions from the aqueous phase by Zn‐O bonds between Zn^2+^ and the carboxylic acid group. Because of the flexible OA chains, the as‐synthesized ZOmAF‐OA sheets displayed a certain degree of flexibility and were easy to deform. When the temperature was increased, these flexible ZOmAF‐OA sheets tended to bend or curl. Meanwhile, OA molecules tended to assemble via hydrophobic interactions among their hydrophobic tails to form micelles. The micellization of OA molecules provided the driving force for the self‐scrolling of ZOmAF‐OA sheets, producing 1D ZOmAF‐OA scrolls and nanotubes. The surface areas of ZOmAF‐OA sheets and ZOmAF‐OA nanotubes were analyzed by measuring N_2_ adsorption‐desorption isotherms at 77 K. As shown in Figure S6 (Supporting Information), the ZIF‐8 sample had a BET surface area of ≈1108.6 m^2^ g^−1^, and the ZIF‐L sample had a lower BET surface area of ≈199.5 m^2^ g^−1^, which was consistent with previously reported values. The ZOmAF‐OA sheets had a BET surface area of ≈145.3 m^2^ g^−1^, and the ZOmAF‐OA nanotube had a BET surface area of ≈198.2 m^2^ g^−1,^ which was comparable to the surface area of ZIF‐L nanosheets. This result indicates that the synthesized ZOmAF‐OA sheets have limited microporosity, which could be attributed to the blockage by the long carbon chain OA decoration. Additionally, the layered arrangement limits the available surface area and pore volume compared to the 3D interconnected MOFs like ZIF‐8.

**Figure 3:**
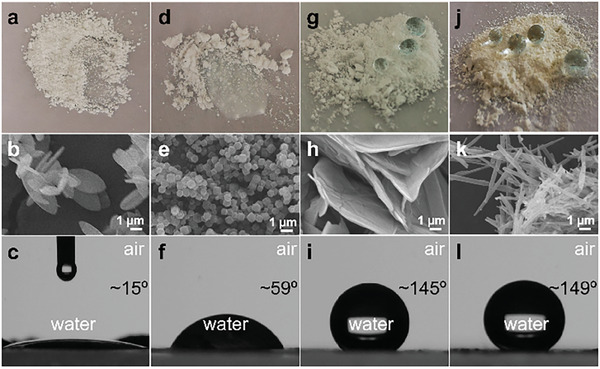
Surface properties of different ZIFs and the synthesized ZOmAF‐OA structures. a) A photo showing ZIF‐L powder wetted by water drops; b) SEM image of the ZIF‐L showing 2D leaf‐like morphology; c) water contact angle (WCA) of the ZIF‐L powder; d) a photo showing ZIF‐8 powder wetted by water drops; e) SEM image of the ZIF‐8 showing 3D truncated dodecahedra morphology; f) WCA of the ZIF‐8 powder; g) a photo showing spherical water drops stay on ZOmAF‐OA powder prepared by interfacial synthesis at room temperature; h) SEM image showing 2D layered morphology of the ZOmAF‐OA; i) WCA of the 2D ZOmAF‐OA powder; j) a photo showing water drops stay on ZOmAF‐OA powder prepared by interfacial synthesis at 50 °C for 48 h; k) SEM image showing 1D nanotube morphology of the ZOmAF‐OA; l) WCA of the 1D ZOmAF‐OA powder.

As discussed above, OA molecules are linked to the interfacially synthesized ZOmAF‐OA structures by forming Zn‐O bonds between zinc centers and the hydrophilic carboxylic acid head of OA molecules. In this case, hydrophobic carbon tails of OA molecules were expected to cover the ZOmAF‐OA surfaces, forming a hydrophobic shell layer. To confirm this, the surface wetting properties of the as‐synthesized ZOmAF‐OA products were investigated by performing water contact angle measurements, which were compared with those of ZIF‐L and ZIF‐8. As shown in **Figure** **3**a–c, ZIF‐L powder with a 2D leaf‐like morphology was easily wetted by water drops and had a WCA of ≈15° in air, suggesting a highly hydrophilic surface property. The ZIF‐8 sample with a 3D truncated dodecahedra morphology was also easily wetted by water drops and showed a WCA of ≈59° in air (Figure 3d–f), suggesting a hydrophilic surface property.

The hydrophilic surface properties of ZIF‐L and ZIF‐8 make them potentially sensitive to water or moisture content in practical applications. Interestingly, the ZOmAF‐OA sheets synthesized in this work showed excellent hydrophobicity, with water drops remaining spherical on the powder and a WCA of ≈145° in air (Figure 3g–i). Likewise, the 1D ZOmAF‐OA nanotubes also exhibited fascinating hydrophobic surface properties with a WCA of ≈145° in air (Figure 3j–l). The hydrophobic properties of both the ZOmAF‐OA sheets and 1D ZOmAF‐OA nanotubes remained unchanged for a long time. For example, they were observed floating on the water surface for up to 30 days (Figure S7, Supporting Information). The superior hydrophobic surface properties of ZOmAF‐OA sheets and nanotubes suggest their excellent water/moisture stability, which is significant for a wide range of real‐world industrial applications, such as gas separation, catalysis, and liquid separations.

**Figure 4:**
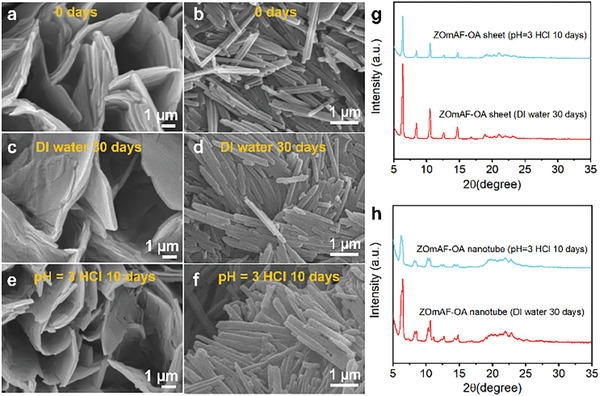
Stabilities of ZOmAF‐OAs in water conditions. SEM images of a) original ZOmAF‐OA sheets, b) ZOmAF‐OA nanotubes, c) ZOmAF‐OA sheets, and d) ZOmAF‐OA nanotubes after soaking in deionized (DI) water for 30 days; e) ZOmAF‐OA sheets, and f) ZOmAF‐OA nanotubes after soaking in pH = 3 HCl solution for 10 days. g) XRD patterns of ZOmAF‐OA sheets after stability tests. h) XRD patterns of ZOmAF‐OA nanotubes after stability tests.

To confirm the enhanced stability of the as‐synthesized ZOmAF‐OAs, a water stability experiment was performed by soaking different ZIF powder samples (ZIF‐L, ZIF‐8, 2D ZOmAF‐OA, 1D ZOmAF‐OA) in deionized (DI) water for up to 30 days. The morphology variation as well as the structural change of the tested ZIFs after soaking were monitored by SEM and XRD characterizations. The SEM observations indicated that the ZIF‐L sample completely transformed into an amorphous phase after 7 days (Figure S8, Supporting Information). Similarly, the ZIF‐8 sample started to transform into an amorphous phase after 7 days in water (Figure S9, Supporting Information). These results indicated that hydrophilic ZIF‐L and ZIF‐8 samples both have insufficient long‐term stability in a water environment. In comparison, ZOmAF‐OAs exhibited greatly enhanced water stability. As shown in **Figure** **4**, the morphology of both ZOmAF‐OA sheets and 1D ZOmAF‐OA nanotubes remained nearly unaffected after being soaked in water for up to 30 days (Figure 4a–d). The exceptional water stability of ZOmAF‐OAs was attributed to their highly hydrophobic nature, which provided the metal‐ligand bond with a shield against attack from water molecules. Furthermore, the chemical stability of different ZIFs was determined by soaking them in solutions with different pH values. As shown in Figure 4e,f, ZOmAF‐OAs were able to maintain their original morphology after being soaked in the pH = 3 HCl solution for up to 10 days. XRD analysis further confirmed that their crystalline structures remained intact after these chemical stability tests (Figure 4 g,h). This indicates that the ZOmAF‐OAs synthesized in this work possess superior chemical stability under harsh acidic conditions. However, ZOmAF‐OAs were found to have insufficient stability in harsh alkaline solutions, with their morphologies changed after being soaked in a pH = 12 NaOH solution for 24 h (Figure S10, Supporting Information). For comparison, the chemical stabilities of ZIF‐L and ZIF‐8 were also investigated. SEM and XRD results indicated that the structures of ZIF‐L and ZIF‐8 were destroyed after soaking in pH = 3 HCl solution and pH = 12 NaOH solution for 24 h (Figures S11,S12 and Table S5, Supporting Information).

**Figure 5:**
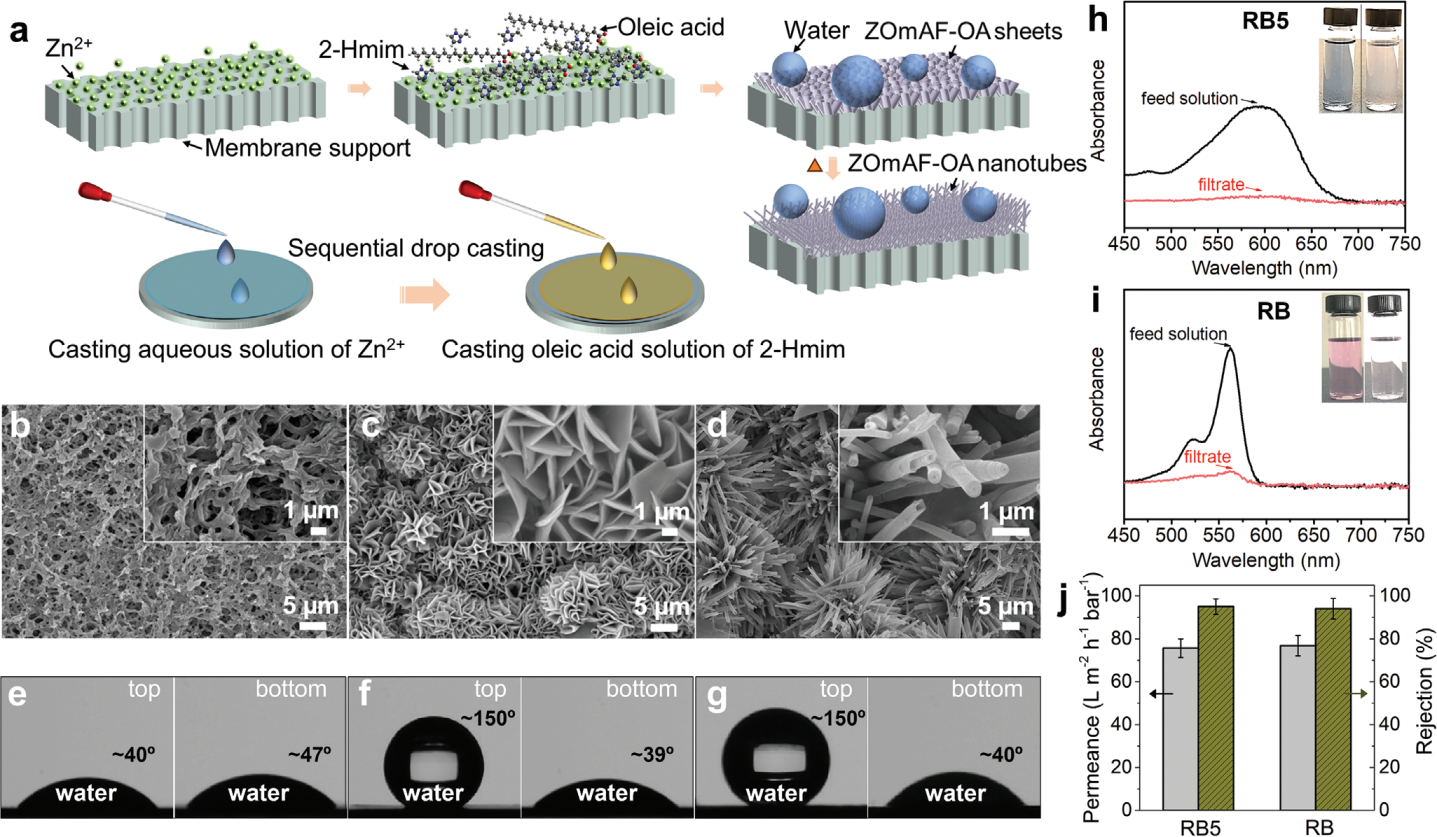
a) Schematic illustration showing the fabrication of ZOmAF‐OA‐coated membranes by a simple sequential drop casting process; SEM images of b) the bare nylon membrane surface, c) the membrane surface prepared by sequential drop casting at room temperature, and d) the membrane surface prepared by sequential drop casting at 50 °C; WCA of the top and bottom surfaces of e) the bare nylon membrane, f) the ZOmAF‐OA sheet‐coated membrane, and g) the 1D ZOmAF‐OA nanostructure‐coated membrane; UV‒Vis spectra of 5 mg L–1 reactive black 5 (RB5) solution (h) and rose Bengal (RB) solution (i) before and after filtration through the ZOmAF‐OA‐coated membrane. The insets show corresponding photos of the feed and filtrate solutions. j) Permeance and rejection of the ZOmAF‐OA‐coated membrane for reactive black 5 and rose bengal molecules.

The excellent stability of ZOmAF‐OA structures in water conditions indicates that they have great potential to be used in a range of applications. In this work, the application of ZOmAF‐OAs in liquid separation was demonstrated as a proof‐of‐concept study. A simple sequential drop‐casting method was developed to prepare ZOmAF‐OA‐coated porous membranes, as presented in the diagram in **Figure** **5**a. A porous nylon membrane with a pore size of ≈200 nm and a diameter of ≈47 mm was employed as the support. Prior to the casting process, the nylon membrane was immersed in Zn(NO_3_)_2_.6H_2_O aqueous solution (1 M) for 1 h. Then, the aqueous solution of Zn(NO_3_)_2_.6H_2_O was preferentially cast on the nylon membrane surface, followed by an OA solution containing 2‐methylimidazole ligands. Next, the membranes were cured for 12 h at room temperature and 50 °C to obtain different ZOmAF−OA structures on the membrane surfaces. The surface morphologies of the membrane surfaces were observed using SEM. As shown in Figure 5b, the pristine nylon membrane substrate showed a porous, rough surface. After the sequential drop‐casting process and curing at room temperature, the membrane surface was uniformly covered by a layer of ZOmAF‐OA sheets (Figure 5c). After being cured at 50 °C, the membrane surface was covered by 1D ZOmAF‐OA nanostructures (Figure 5d). SEM observation results confirmed that this simple sequential drop‐casting method was effective for preparing ZOmAF‐OA‐coated membranes. The wetting properties of the membrane surfaces were determined by performing WCA measurements. As shown in Figure 5e, the pristine porous nylon membrane showed hydrophilic surfaces on both sides, with WCAs of ≈40° and ≈47°, respectively. After the sequential drop casting process, the ZOmAF‐OA‐coated membranes exhibited asymmetric wettability, with one surface showing superhydrophobic properties (WCA of up to ≈150°) and the other surface showing hydrophilic properties (WCA of ≈40°), as presented in Figure 5f,g. Such an asymmetric wettability indicated that the ZOmAF‐OA‐coated membranes exhibited Janus wetting properties.

The membrane was applied for molecular separation in an organic solvent (methanol). ZOmAF‐OA sheet‐coated membranes were selected for the separation tests because they showed more uniform ZOmAF coverage than ZOmAF‐OA nanotube‐coated membranes. The permeance of the membrane was determined by calculating the volume of methanol permeate per unit time. Meanwhile, the separation performance of the membrane was estimated using different organic dye molecules, including rose bengal (RB, MW = 1017.6 g mol^−1^) and reactive black 5 (RB5, MW = 992 g mol^−1^). The concentration of dye molecules in the solutions before and after the filtration tests was estimated by UV‒Vis spectroscopy. The ZOmAF‐OA‐coated membrane was effective for separating organic dye molecules such as RB5 (Figure 5h) and RB (Figure 5i) from methanol. The membrane permeances, as well as calculated dye rejections, are presented in Figure 5j. The ZOmAF‐OA‐coated membrane exhibited ≈95.2 ± 3.8% rejections for reactive black 5 and rose bengal molecules in methanol, with permeances of ≈75 ± 5 L m^−2^ h^−1^ bar^−1^. The results were comparable to those of ZIF‐intercalated graphene oxide membranes, although the latter are much more permeable.^[^ ^36^ ^]^ However, further effort to prepare even thinner high‐quality, hydrophobic ZOmAF‐OA‐coated membranes is the key to improving membrane permeability.

## 3 Conclusions

In summary, we designed an oil/water interfacial synthesis method to prepare new ZOmAF nanostructures, denoted as ZOmAF‐OAs. 2D ZOmAF‐OA sheets were obtained through the interfacial synthesis method at room temperature. 1D ZOmAF‐OA nanotubes were obtained through the self‐scrolling of ZOmAF‐OA sheets. We found that the self‐scrolling of ZOmAF‐OA sheets was driven by the micellization of amphiphilic OA molecules. The synthesized ZOmAF‐OAs showed superhydrophobic properties with water contact angles up to 150°. More importantly, they demonstrated exceptional stability under a water environment with morphologies unaffected after being soaked in deionized water for up to 30 days and in pH = 3 HCl solution for up to 10 days. In addition, a simple sequential drop‐casting method was developed to prepare ZOmAF‐OA‐coated membranes. The prepared ZOmAF‐OA‐coated membranes showed Janus surface‐wetting properties. TheZOmAF‐OA‐coated membrane was effective for molecular separation with rejection rates of ≈90% for organic dye molecules. Self‐scrolling of 2D ZOmAF‐OAs was realized to produce 1D ZOmAF‐OAs for the first time. We established a new interfacial synthesis approach to prepare MOmAF with superior stability in aqueous conditions. We expect that the interfacial synthesis concept developed herein can be applied to design further examples of water‐stable MOmAF. However, it is worth mentioning that the interfacial synthesis approach gave a low yield of < 5 wt.%. A common solution‐based method was also applied to synthesize ZOmAF‐OAs, which gave a considerably higher yield of 40.7 ± 3.5 wt.% comparable to most MOF and ZIF syntheses (see **1.2.1. Synthesis of ZOmAF‐OAs** and Figure S13 (Supporting Information) for the procedures, recipe, and characterizations of ZOmAF‐OA samples prepared via the solution‐based method).^[^ ^37^ ^]^ A future work is required to further understand the structural properties of ZOmAF‐OAs (1D and 2D) and their applications in various fields, including but not limited to gas adsorption/separation, catalysis, and liquid separation.^[^ ^38^ ^]^

## Conflict of Interest

The authors declare no conflict of interest.

## Author Contributions

T.C. and X.W. contributed equally to this work. The manuscript was written through the contributions of the authors. All authors have approved the final version.

## Supporting information

Supporting Information

## Data Availability

The data that support the findings of this study are available on request from the corresponding author. The data are not publicly available due to privacy or ethical restrictions.
