# Supplementary material for: New 2D Metal‐Organic Monoacid Framework (MOmAF): Realization of Extreme Water Repellence
Source: Small. 2024 Jul 31;20(50):2404224. doi: 10.1002/smll.202404224 (PMC11636070; doi:10.1002/smll.202404224)
Supplement: Supplementary file 1 — Supporting Information [file SMLL-20-2404224-s001.docx]

**New two-dimensional metal-organic monoacid framework (MOmAF): realization of extreme water repellence**

Ting Chen,^[a,†]^ Xiuming Wei,^[a,†]^ Thomas Fabiani,^[a]^ Baoyu Liu,*^[b]^ Yaohao Yang,^[a]^ Allana Lewis,^[a]^ Nurul A. Mazlan,^[a]^ Fraz Saeed Butt,^[a]^ Siyu Chen,^[a]^ Qinfeng Gu,^[c]^ Norbert Radacsi,^[a]^ Huanting Wang,^[d]^ Maria Grazia De Angelis,^[a]^ Shuiqing Yang,*^[e]^ Haiqun Chen,*^[f]^ and Yi Huang*^[a]^

[a] Dr. T. Chen, X. Wei, T. Fabiani, Y. H. Yang, Dr. A. Lewis, N. A. Mazlan, Dr. F. S. Butt, S. Chen, Dr. N. Radacsi, Prof. M. G. De Angelis, Dr. Y. Huang
School of Engineering, Institute for Materials & Processes, The University of Edinburgh
Robert Stevenson Road, EH9 3FB, United Kingdom

E-mail: [Yi.Huang@ed.ac.uk](mailto:Yi.Huang@ed.ac.uk)

[b] Dr. B. Y. Liu

School of Chemical Engineering and Light Industry, Guangdong Provincial Key Laboratory of Plant Resources Biorefinery, Guangzhou Key Laboratory of Clean Transportation Energy Chemistry, Guangdong University of Technology, 510006 Guangzhou, China

Jieyang Branch of Chemistry and Chemical Engineering Guangdong Laboratory (Rongjiang Laboratory), Jieyang 515200, China

E-mail: [baoyu.liu@gdut.edu.cn](mailto:baoyu.liu@gdut.edu.cn)

[c] Dr. Q. F. Gu

Australian Synchrotron

Clayton, Melbourne, Victoria 3168 (Australia)

[d] Prof. H. T. Wang

Department of Chemical and Biological Engineering, Monash University

Clayton, Victoria 3800, Australia

[e] Mr S. Q. Yang

Jiangsu Dingying New Materials Co., Ltd., Changzhou, Jiangsu, 213031, China.

E-mail: y.yang-331@sms.ed.ac.uk

[f] Prof. H. Q. Chen

Key Laboratory of Advanced Catalytic Materials and Technology, Advanced Catalysis and Green Manufacturing Collaborative Innovation Center, Changzhou University, Changzhou, Jiangsu Province 213164, China.

E-mail: [chenhq@cczu.edu.cn](mailto:chenhq@cczu.edu.cn)

[†] These authors contributed equally to this work.

Table of Contents

1. Experimental Procedures …………………………………………………………………………………………..3

2. Supporting Figures…………………………………………………………………………………………………..5

3. Supporting Tables.…………………………………………………………………………………………………19

4. References………………………………………………………………………………………………………….20

1. Experimental Procedures

**1.1. Materials**

Zinc nitrate hexahydrate (Zn(NO_3_)_2_·6H_2_O; 98%) and 2-methylimidazole (2-Hmim; 99%) were purchased from Alfa Aesar. Oleic acid was obtained from Sigma‒Aldrich Company Ltd. Hydrochloric acid (HCl, ~37%), and sodium hydroxide (NaOH, ≥97%). A nylon membrane (47 mm diameter, 0.2 µm pore size) was ordered from Whatman, Inc. rose bengal (C_20_H_2_Cl_4_I_4_Na_2_O_5_), and reactive black 5 (C_26_H_21_N_5_Na_4_O_19_S_6_) were obtained from Alfa Aesar. All chemicals were used as received.

**1.2. Methods**

**1.2.1. Synthesis of ZOmAF-OAs**

1. **Interfacial assembly method**

Hydrophobic two-dimensional ZOmAF-OAs were synthesized through a simple oil/water interfacial assembly method. Typically, 5 mL of aqueous solution of Zn(NO_3_)_2_·6H_2_O with a concentration of 1 M was first added to a petri dish (with a diameter of ~60 mm). Then, 1 mL of 2-methylimidazole dissolved in oleic acid at a concentration of 0.4 M was added dropwise to the surface of the Zn(NO_3_)_2_·6H_2_O solution in the petri dish. The two-phase mixture was aged for ~24 h at room temperature to obtain two-dimensional ZOmAF-OA layers. For the preparation of 1D ZOmAF-OA nanoscrolls and nanotubes, the two-phase mixture was aged for 12 h ~ 48 h at 50 °C in an oven. The product was collected by centrifugation at ~5000 rpm for 10 min, followed by a methanol wash. After repeating the centrifugation process three times, the final product was dried in an oven at 50 °C for further characterization.

1. **Solution-based synthesis**

A solvothermal synthesis method was also used to synthesize ZOmAF-OA at room temperature using a molar ratio of 1 Zn(NO_3_)_2_: 6 H_2_O: 1.187 2-Hmim: 1.278 OA: 148.223 MeOH. Typically, 4g Zn(NO_3_)_2_·6H_2_O (≥99.0%, Sigma-Aldrich) and 1.31g 2-methylimidazole (2-Hmim, 99%, Sigma-Aldrich) were dissolved in 40 mL of methanol separately and then mixed. After 2 min, 6 mL of oleic acid (OA, 90%, Sigma-Aldrich) was added to the mixture. The mixture was stirred at 500 rpm, 25 °C for 24 h. Powders were washed with methanol and then dried in the oven at 50 °C for 18 h for further characterization.

The mass yield of ZOmAF-OA was roughly calculated according to the following equation:

$$yield=\frac{m_{product}}{m_{reactants}}\times100\%$$

*where m_product_* is the weight of ZOmAF-OA and *m_reactants_* is the weight of Zn(NO_3_)_2_·6H_2_O and oleic acid.

According to the equation above, the yields of the 2D ZOmAF-OA nanosheet and 1D ZOmAF-OA nanotube are 4.72 ± 0.51 wt% and 4.40 ± 0.38 wt%, respectively. However, considering the ZOmAF-OA crystallization only occurs at the interfaces, very limited Zn(NO_3_)_2_·6H_2_O and oleic acid were consumed. While the majority of the reactants remained in each solution. In contrast, the yield of the ZOmAF-OA sheet is 40.73 ± 3.52 wt% (based on 8 syntheses), which is significantly higher than that of the Interfacial assembly method but is comparable with normal ZIF/MOF synthesis.

**1.2.2. Preparation of ZIF-L and ZIF-8**

In this work, ZIF-L and ZIF-8 were prepared for comparison with the ZOmAF-OAs synthesized in this work. ZIF-L was prepared according to a previously reported method. ^[1]^ In brief, aqueous solutions of Zn(NO_3_)_2_·6H_2_O (0.59 g, 40 mL) and 2-methylimidazole (1.30 g, 40 mL) were mixed (V:V = 1:1) and stirred for 2 h at room temperature (~25 °C). Then, the powder product was collected by centrifugation at ~5000 rpm for 10 min, followed by ethanol wash. After repeating the centrifugation and rinsing process three times, the final product was dried in an oven at 50 °C for further characterization. The ZIF-8 sample was prepared by adding ethanol to the ZIF-L synthesis solution. ^[2]^ Typically, aqueous solutions of Zn(NO_3_)_2_·6H_2_O (0.59 g, 20 mL) and 2-methylimidazole (1.30 g, 20 mL) and 10 mL of ethanol were mixed and stirred for 1 h at room temperature (~25 °C). Then, the powder product was collected by centrifugation at ~5000 rpm for 10 min, followed by ethanol wash. After repeating the centrifugation and rinsing process three times, the final product was dried in an oven at 50 °C for further characterization.

**1.2.3. Fabrication of the ZOmAF-OA-coated membrane**

A simple sequential drop casting method was developed to fabricate Janus ZOmAF-OA-coated membranes with asymmetric wettability. Typically, a piece of porous nylon membrane with a diameter of 47 mm and pore size of ~200 nm was first soaked in an aqueous solution of Zn(NO_3_)_2_·6H_2_O with a concentration of 1 M. Then, ~1 mL of the Zn(NO_3_)_2_·6H_2_O solution was cast on the surface of the nylon membrane. Then, ~0.2 mL oleic acid solution of 2-methylimidazole was cast onto the membrane surface. The membrane was aged for ~12 h at room temperature and 50 °C to obtain a ZOmAF-OA sheet-coated membrane and a 1D ZOmAF-OA nanostructure-coated membrane, respectively.

**1.2.4 Simulation methodology**

Simulations were run in LAMMPS [3]. Two identical slabs were placed at a distance of 50 Å between the Zn atoms. The oleic fatty acid was sketched in cis-conformation. The unit cell was extended to reach a size of at least twice the cutoff in every direction. Open Babel GUI 3.1.1 [3] was used for format conversion of the topology files. Atom types were assigned using the toolkit lammps-interface [4], considering the periodic image involved in the non-bonded interactions. UFF [5] was used as a force field. Non-bonded interactions were modelled with a pairwise 6-12 Lennard Jones potential, using a geometric mixing rule, and the cut off was set at 12.5. The simulation started with a minimization phase (maximum iterations equal to 10^6^ and stopping tolerance for energy 10^–7^ and stopping tolerance for force 10^–8^). Equilibration and production phases were NPT molecular dynamics simulations. Periodic boundary conditions were applied. Nose-Hoover thermostat was set at 298 K (damping factor equal to 10). The isotropic barostat was set to 1 atmosphere (damping factor equal to 1000). Production was run for at least 500 ps after equilibration of total energies and its components were observed. Simulations at 298 K were run in duplicate, using a different seed on the initial velocity assignment. Different heating schemes were used to prove the independence of results from the cooling path. Distance between slabs was calculated as the closest peak in the radial distribution function between zinc atoms in different slabs, considering minimum image convention. Data analysis was carried out in Python [4]. Simulations were run on Eddie, the supercomputing cluster of The University of Edinburgh [5] and on Precision 5820 Tower XCTO Base (Intel Core i9-10980XE 3.0 GHz, 4.8 GHz Turbo, 18C, 24.75 MB Cache, HT).

**1.2.5. Dye molecule separation experiment**

The dye molecule rejection performance of the ZOmAF-OA-coated membrane was evaluated using a vacuum filtration apparatus under a vacuum pressure of ~0.6 bar. The permeance (F) of the membrane was obtained by calculating the volume of permeate in unit time, according to the equation F = V/(A • ∆t • ∆P), where V (L) is the volume of permeate, A (m2) is the effective filtration area, Δt (h) is the filtration time, and ΔP is the applied pressure. The rejection of the membrane for dye molecules was evaluated with 20 mL of the feed solution filtering across the membrane. The concentration of dye molecules was determined using a UV‒Vis spectrophotometer (Thermo Scientific Evolution 60). The dye rejection (R) was calculated according to the equation R = (1 − C_p_/C_f_) × 100%, where C_p_ is the concentration of dyes in the permeate, and C_f_ is the concentration of dyes in the feed solution.

**1.2.6. Characterizations**

The surface morphology of the prepared materials was characterized by a JEOS JSM-IT100 scanning electron microscope (SEM). Contact angle (CA) measurements were performed using the Ossila contact angle goniometer. Fourier transform infrared spectroscopy (FTIR) spectra were obtained on a Nicolet iS10 instrument. Zn K-edge X-ray absorption near edge structure (XANES) spectra and extended X-ray absorption fine structure (EXAFS) spectra were collected in transmission mode at room temperature to evaluate the chemical state and configuration of Zn. The process of data reduction and analysis, as well as the fitting of the EXAFS spectra, were executed utilizing the Athena and Artemis modules within the Demeter data analysis suite, which in turn leverages the capabilities of the FEFF6 program for EXAFS data fitting. For energy calibration purposes, both the standard and Zn foil were employed; the latter, serving as a reference, was measured concurrently. The crystalline structure of the synthesized ZIF-L on the oil/water interface was confirmed using an X-ray diffractometer (XRD, Bruker D8 Advance) with Cu Kɑ radiation in a 2θ range of 4.0° ~ 40.0°. N_2_ adsorption-desorption isotherms were obtained on a Quantachrome Autosorb IQ instrument. Elemental analyses were performed on at least 10 samples using Inductively Coupled Plasma Spectroscopy (ICP-OES: Agilent 5110) and Elemantar (Vario EL cube).

2. Supporting Figures


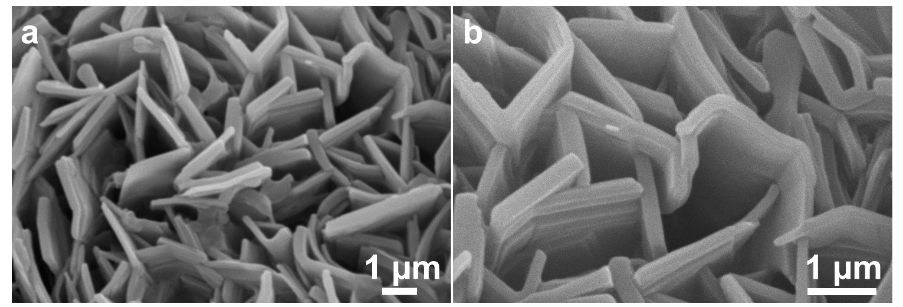


**Figure S1.** SEM images of the interfacial synthesized ZOmAF-OA sheets under different magnifications.


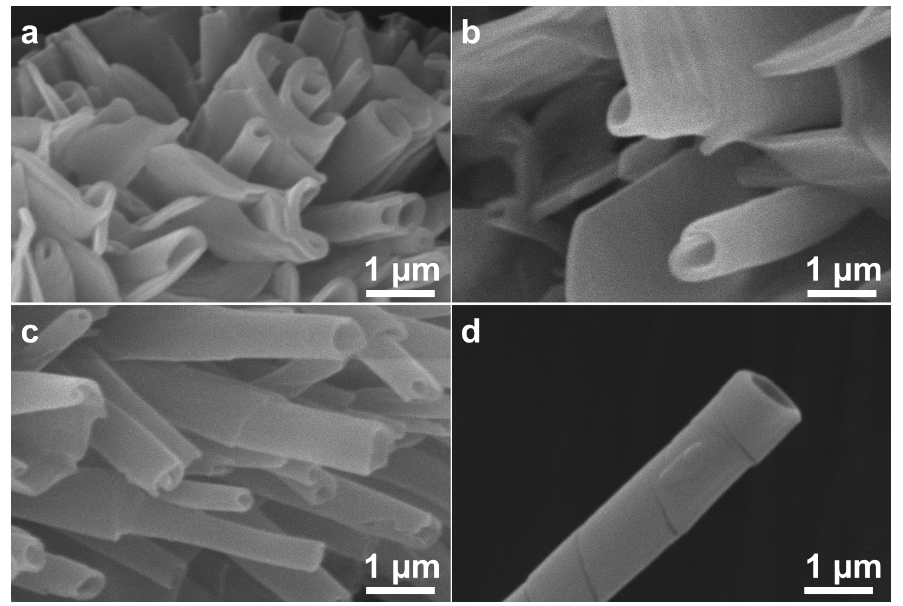


**Figure S2.** SEM images of (a), (b) the self-scrolling ZOmAF-OA sheets; (c), (d) the produced 1D ZOmAF-OA scrolls.


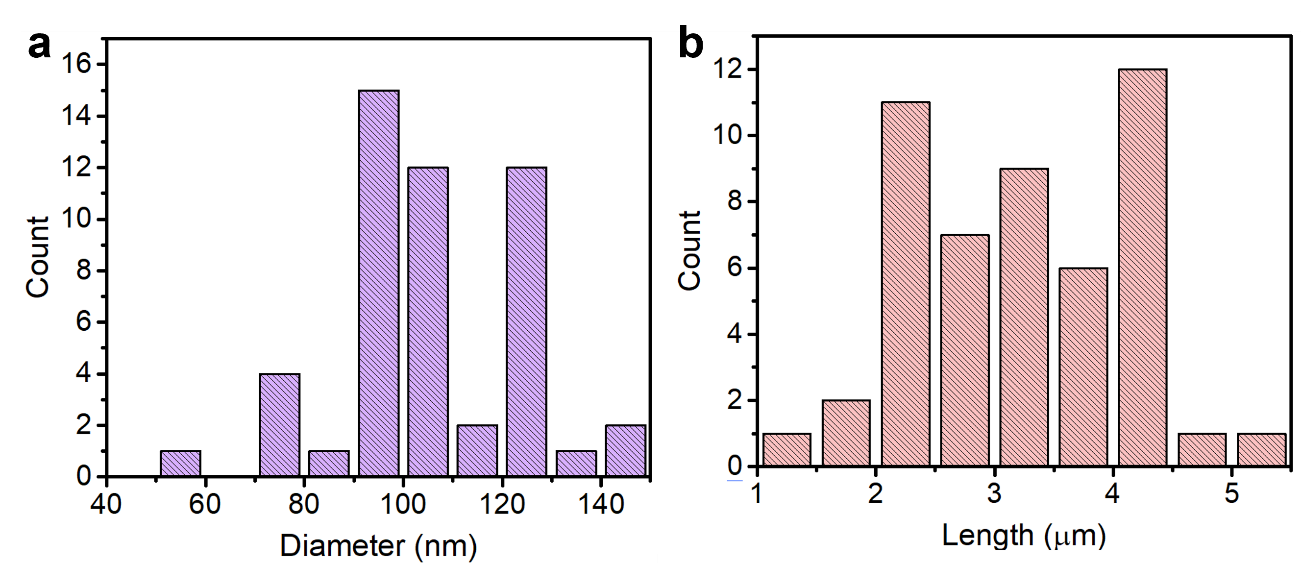


**Figure S3.** The estimated size distribution of (a) the diameter and (b) the length of the ZOmAF-OA nanotubes synthesized at 50 °C for 48 h.


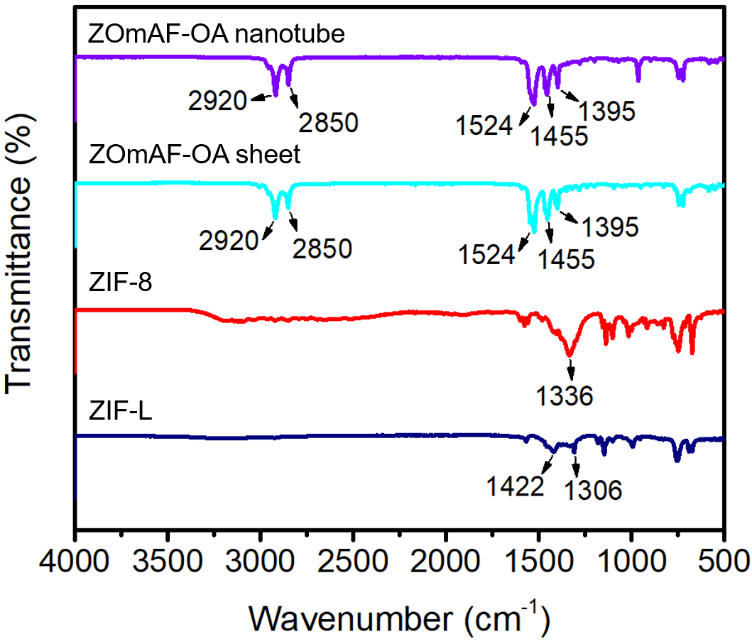


**Figure S4.** FTIR spectra of ZIF-L, ZIF-8, and the synthesized ZOmAF-OA sheet as well as the ZOmAF-OA nanotube.

**
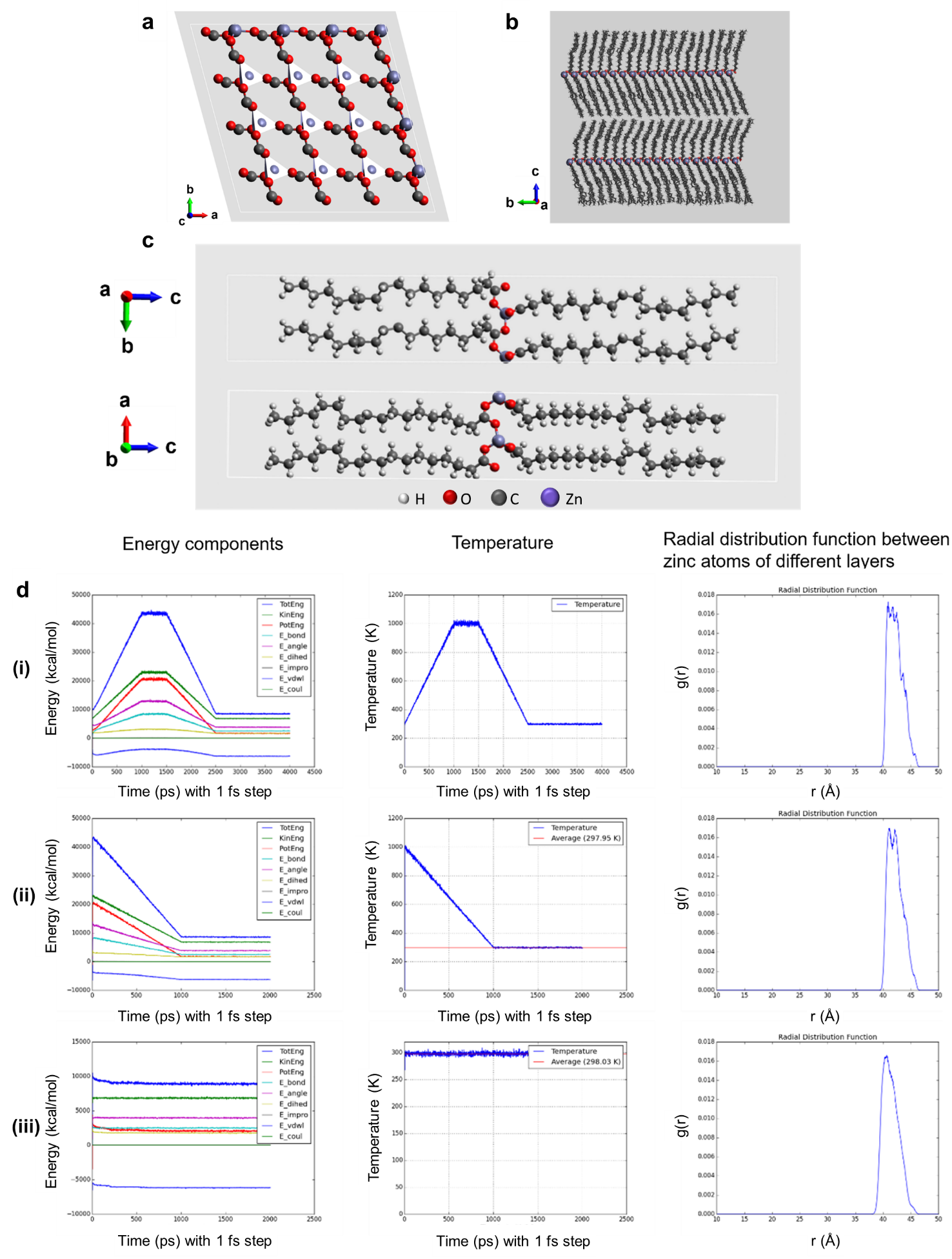
**

**Figure S5.** Schematic illustration of the synthesized ZOmAF-OA sheet structure viewed from different planes. (a) the basal plane (view from *c*, normal to the plane; fatty acids tails are omitted for clarity); (b) the projection of one slab material in the direction *a* and *b*; (c) the two ZOmAF-OA layers at equilibrium distance (snapshot of the simulation at the end of the production phase); (d) three different heating-cooling paths showed to not influence the final distance between layers

The structure sketched and simulated is reported in **Figure S5.** The equilibrated structure is based on zinc atoms, laying on the same plane, tetrahedrally coordinating the oxygens by the fatty acids, that are oriented above and below the basal plane. In **Figure S5a,** the fatty acids tails are omitted for clarity and the view is normal to the basal plane. The fatty acids are oriented according to their position with respect to the basal plane, due to the constraint of the tetrahedral coordination (**Figure S5b**); such an ordered arrangement is shown to induce and influence their interaction.

The equilibrated system consists of a pair of ZOmAF-OA layers interacting with each other, bound to their respective periodic image. The layers attract each other, and they reach an equilibrium condition after approximately 500 ps at 298 K. Removing boundary conditions and enlarging the system, e.g. by using a larger number of units, induces no deformation, tilting, or change in the structural behaviour or atomic distances (assessed through radial distribution function and coordinates analysis). Furthermore, different heating and cooling ramps (i–iii in **Figure S5d**) were tested and had no impact on the structure, the d-space, or the order of the organic layers during the production phase. In fact, fatty acids tilt and align along the *a* and *b* directions, according to their position with respect to the basal plane, forming rows of tails, in order to maximize the interactions between them (**Figure S5c**). The variation of the kinetic energy in the system during the heating and cooling phases led to a disruption of the ordered structure of the fatty acids, which is restored when the system is cooled to 298 K, in every simulation. Therefore, despite different kinetic paths followed during the simulation, the tails pack themselves in the same manner. The surface of the ZOmAF-OA layers, assuming a perfect, defect-free coverage, appears to be dense, due to the packing between fatty acids and it does not allow penetration between fatty acid chains of two layers. The equilibrium distance between the basal planes of two different slabs **Figure S5c** (measured as the distance between Zn atoms in different slabs) is equal to 40.85 Å (–3.77% of experimental, equal to 42.44 Å). The mismatch might be related to the different packing of the structure, due to defects and non-ideality of the material in the experimental testing or a limitation in the force field. “

Elemental analyses were performed to get a possible chemical formula for ZOmAF-OA:

(Zn(C_18_H_33_O_2_)_2_)_1–_*_x_*·(Zn(OH)_2_)*_x_* (*x* ≤ 0.15)

Zn(OH)_2_ was found to accompany the growth of ZOmAF-OA. However, its content was in trace amounts (*x*<0.05) for samples synthesized for a longer time and using a solution-based method. More importantly, only trace amounts of N were found in the tests, confirming that no imidazolate was present/coordinated in the structure. As a comparison, the pure ZOmAF-OA structure sketched by molecular simulation showed a formula Zn(O_2_C_18_H_33_)_2_ with a stoichiometric ratio that is in accordance with the oleic acids coordinated by tetrahedral zinc.

The above elemental analyses and molecular simulation results match very well, except that traces of Zn(OH)_2_ were found in the raw samples prepared using interfacial synthesis due to short synthesis time.

**
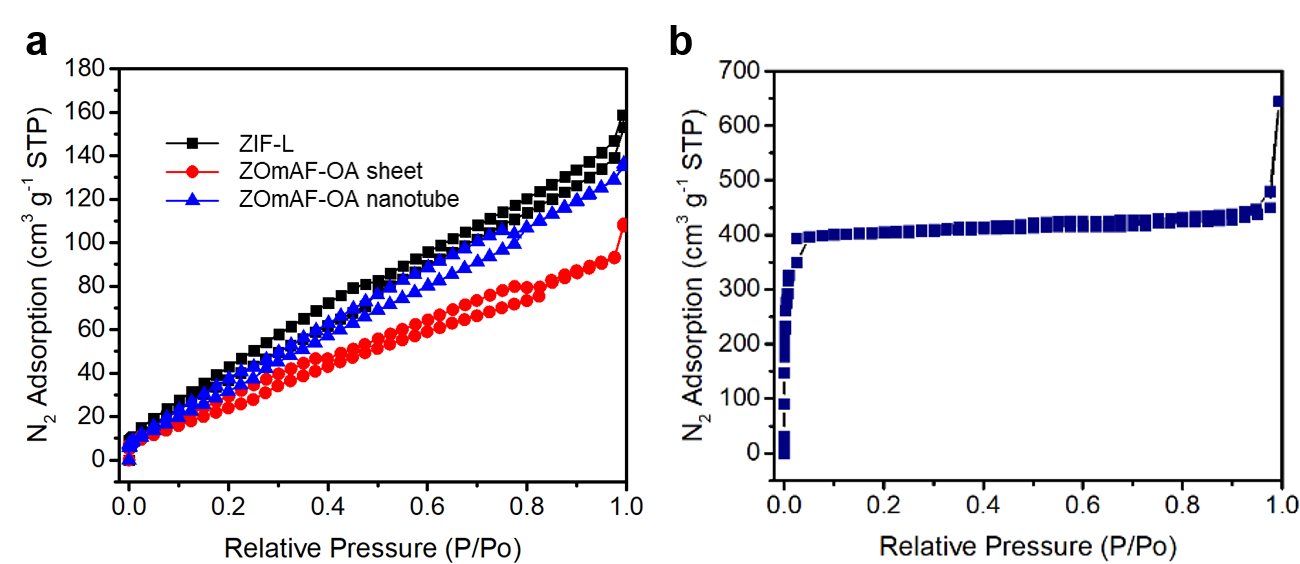
**

**Figure S6.** Nitrogen adsorption-desorption isotherm of (a) ZIF-L, ZOmAF-OA sheet, ZOmAF-OA nanotube, and (b) the ZIF-8 sample.

**
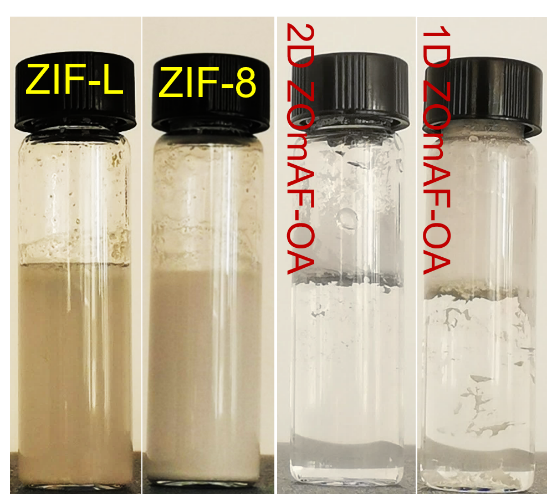
Figure S7.** Pictures of ZIF-L, ZIF-8, ZOmAF-OA sheet, and ZOmAF-OA nanotube powder samples in water after 30 days.


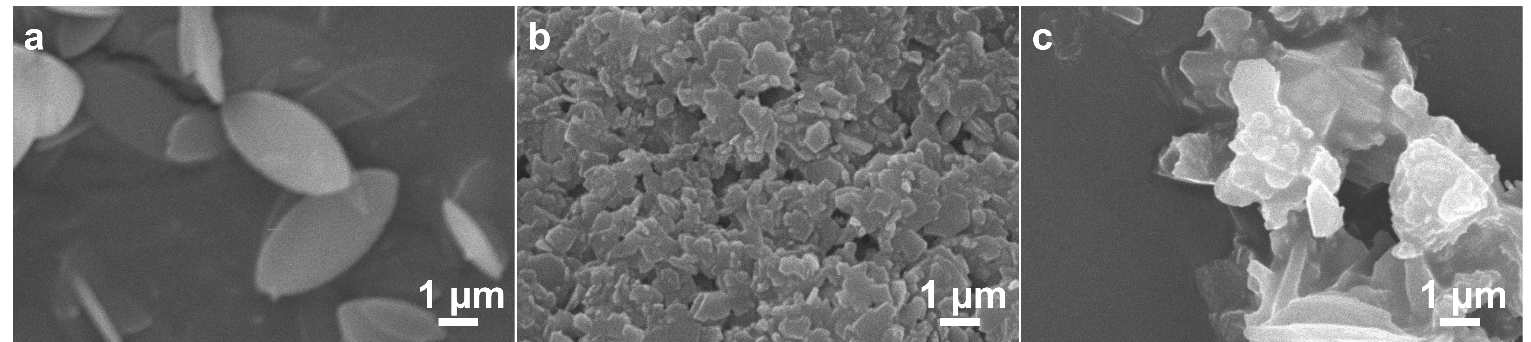
**Figure S8.** SEM images of ZIF-L after being soaked in deionized water for (a) 0 days, (b) 7 days, and (c) 30 days.

**Figure S9.** SEM images of ZIF-8 after being soaked in deionized water for (a) 0 days, (b) 7 days, and (c) 30 days.
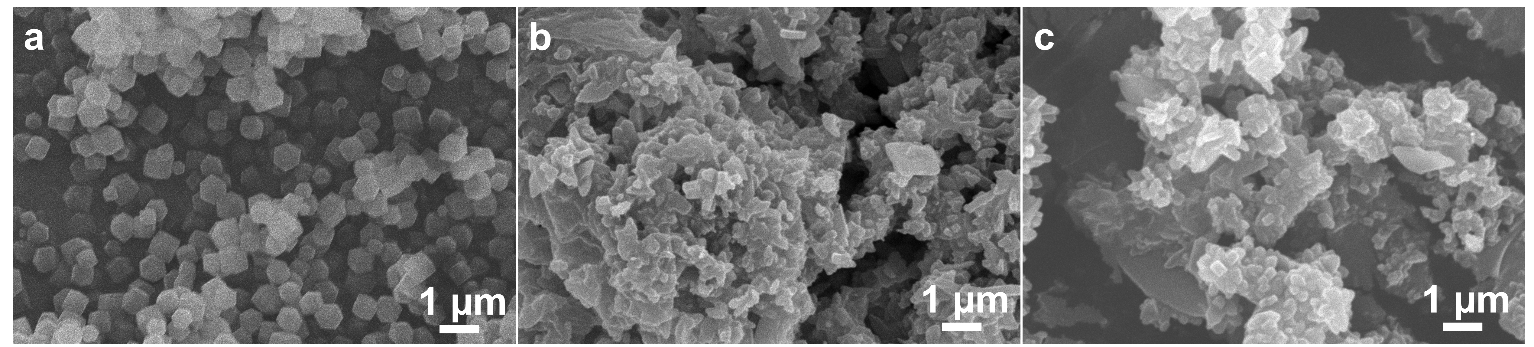


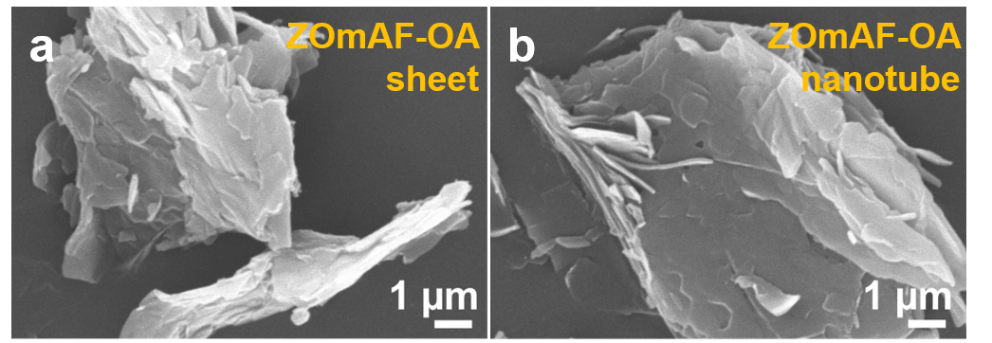


**Figure S10** SEM images of (a) ZOmAF-OA sheets and (b) ZOmAF-OA nanotubes after soaking in pH = 12 NaOH solution for 24 h.


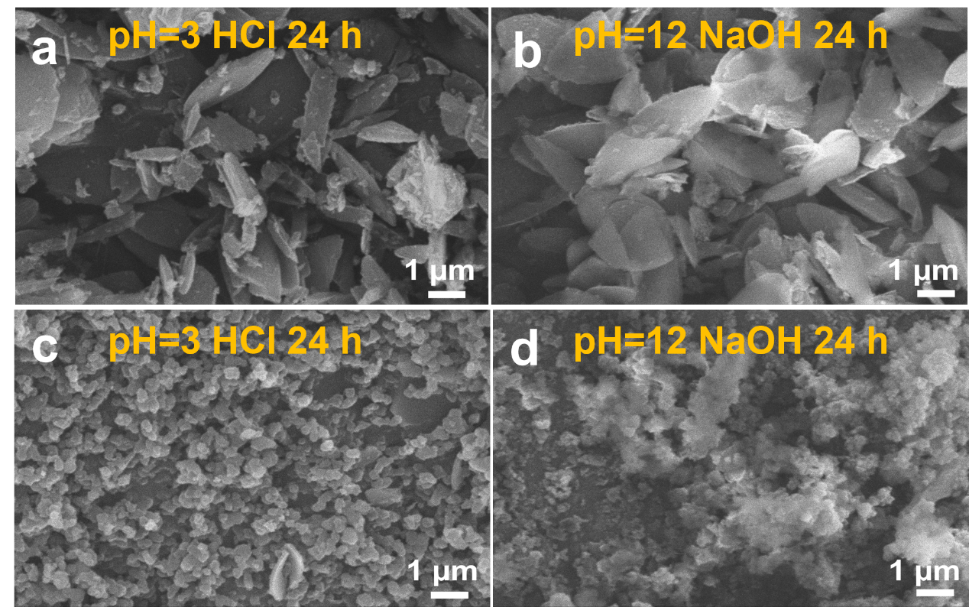


**Figure S11.** SEM images of (a) ZIF-L after soaking in pH = 3 HCl solution for 24 h, (b) ZIF-L after soaking in pH = 12 NaOH solution for 24 h, (c) ZIF-8 after soaking in pH = 3 HCl solution for 24 h, and (d) ZIF-8 after soaking in pH = 12 NaOH solution for 24 h.


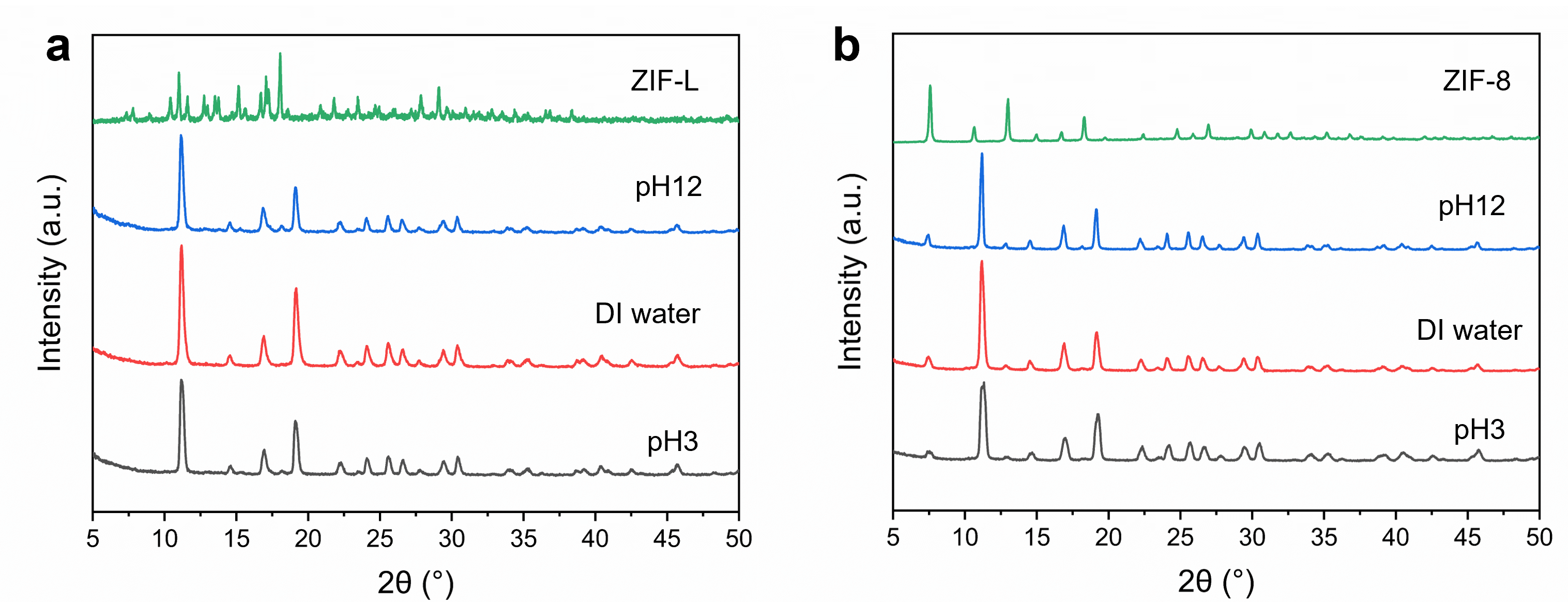


**Figure S12.** PXRD patterns of ZIF-L (a) and ZIF-8 (b) after soaking in pH = 3 HCl solution, DI water, and pH = 12 NaOH solution for 24 h.

As shown in Figure S12, the structure of ZIF-L and ZIF-8 were both destroyed after being soaked for 24 h in DI water, HCl solution (pH = 3), and NaOH solution (pH = 12). PXRD patterns of the treated ZIF-8/ZIF-L suggested that the hydrolysis triggered the formation of new crystalline substance(s). It has been suggested that such phase change could be contributed by the partly replacing of imidazole molecule by hydroxyl group, and produce a possible pseudomorph of ZIF [6][7].

The surface areas of ZIF-L and ZIF-8 were also analyzed by measuring N_2_ adsorption-desorption isotherms at 77 K. The initial ZIF-8 sample had a BET surface area of ~1108.6 m^2^ g^–1^, and the initial ZIF-L sample had a lower BET surface area of ~199.5 m^2^ g^–1^. However, the BET surface area of ZIF-L and ZIF-8 was significantly reduced after being soaked in aqueous media for just 24 h. BET of ZIF-8 was reduced by half after 24 h in water. While in pH = 3 HCl solution and pH = 12 NaOH solution, the BET surface area of ZIF-8 was reduced by more than 90% (see Table S5).


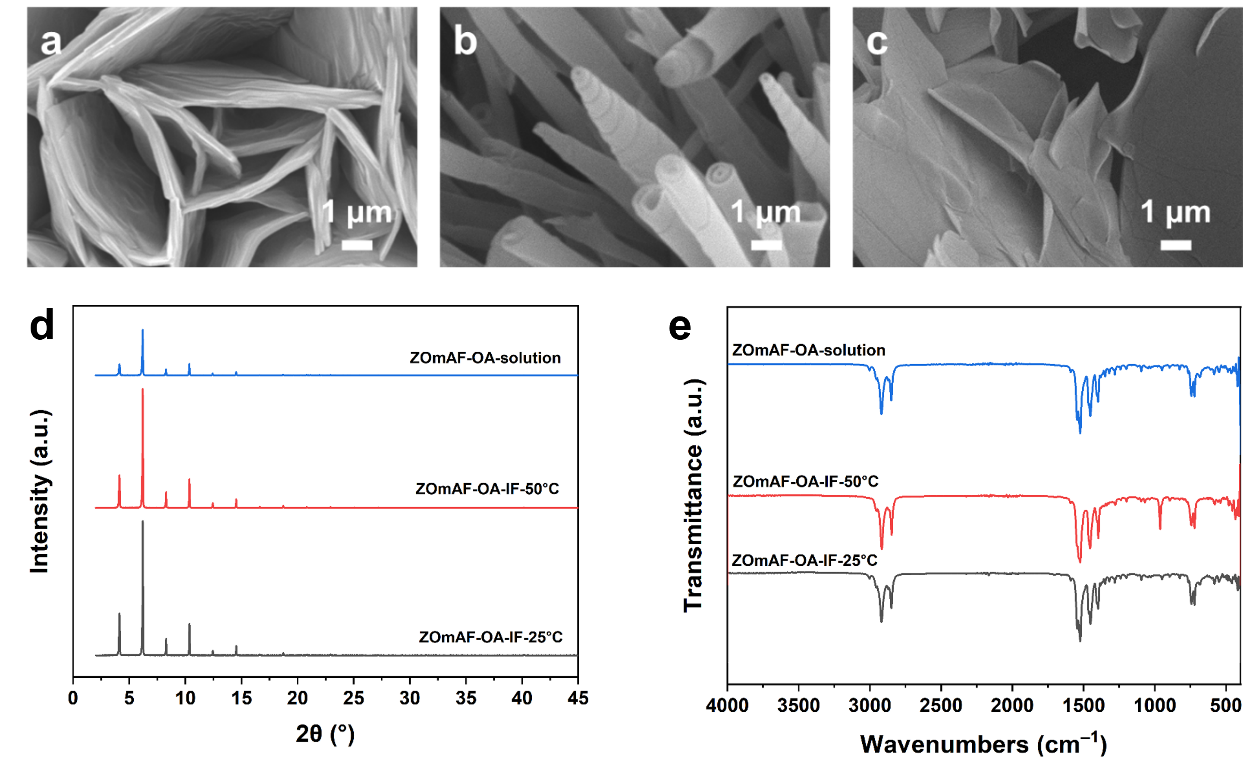


**Figure S13.** SEM images of ZOmAF-OA via different synthesis methods: (a) interfacial synthesis (IF) at 25 °C, (b) 50 °C; and (c) solution-based synthesis at 25 °C. (d) XRD patterns and (e) FTIR spectra of ZOmAF-OAs fabricated by interfacial synthesis and by solution-based synthesis.

As shown in the SEM images (Figure S13a–c), ZOmAF-OA synthesized in a single mixture solution exhibited very similar 2D nanosheet morphology as samples prepared *via* the interfacial synthesis approach. XRD and FTIR results also showed identical spectra for all samples, respectively, confirming that they share the same structure. Therefore, both methods produced the same ZOmAF-OA material. However, the interfacial synthesis might be favourable in ZOmAF membrane preparation, while solution-based synthesis is useful for the scaling up production of ZOmAF powdered materials.

3. Supporting Tables

**Table S1.** FTIR characteristic peak assignments for the ZIF-L sample.

| Wavenumber (cm^-1^) | Assignment |
| --- | --- |
| 1422 | Imidazole ring stretching |
| 1306 | Imidazole ring stretching |
| 1179 | Imidazole ring out-of-plane bending |
| 1146 | Imidazole ring out-of-plane bending |
| 1099 | Imidazole ring out-of-plane bending |
| 753 | Wagging vibration of N–H bonds |
| 689 | Zn–N bonds |
| 672 | Zn–N bonds |
| 420 | Zn–N bonds |

**Table S2.** FTIR characteristic peak assignments for the ZIF-8 sample.

| Wavenumber (cm^–1^) | Assignment |
| --- | --- |
| 1336 | Imidazole ring in-plane bending vibration |
| 1155 | Imidazole ring out-of-plane bending |
| 1137 | Imidazole ring out-of-plane bending |
| 1099 | Imidazole ring out-of-plane bending |
| 747 | Wagging vibration of N–H bonds |
| 672 | Zn–N bonds |
| 420 | Zn–N bonds |

**Table S3.** FTIR characteristic peak assignments for the 2D ZOmAF-OA sample.

| Wavenumber (cm^–1^) | Assignment |
| --- | --- |
| 2920 | –CH_2_– bending vibration |
| 2850 | –CH_2_– bending vibration |
| 670 | Zn–N bonds |
| 580 | Zn–O bonds |
| 550 | Zn–O bonds |
| 420 | Zn–N bonds |
|  |  |
|  |  |
|  |  |
|  |  |

**Table S4.** FTIR characteristic peak assignments for the 1D ZOmAF-OA sample.

| Wavenumber (cm^–1^) | Assignment |
| --- | --- |
| 2920 | –CH_2_– bending vibration |
| 2850 | –CH_2_– bending vibration |
| 580 | Zn–O bonds |
| 480 | Zn–O bonds |
| 458 | Zn–O bonds |
| 435 | Zn–O bonds |
| 420 | Zn–N bonds |
| 411 | Zn–O bonds |

**Table S5.** BET surface area (m^2^ g^−1^) of ZIF-L and ZIF-8 after being soaked in pH = 3 HCl solution, DI water, and pH = 12 NaOH solution for 24 h.

| Conditions  Samples | Original | pH = 3, 24 h | DI water, 24 h | pH = 12, 24 h |
| --- | --- | --- | --- | --- |
| ZIF-L | 199.5 | 20.0 | 21.2 | 45.2 |
| ZIF-8 | 1108.6 | 39.0 | 594.7 | 96.9 |

# 4. References

[1] Z. Zhong, J. Yao, R. Chen, Z. Low, M. He, J.Z. Liu, H. Wang, *J. Mater. Chem. A.* **2015**, *3*, 15715.

[2] A. Lewis, T. Chen, F. S. Butt, X. Wei, N. Radacsi, X. Fan, Y. Huang, *Nanoscale*. **2021**, 13, 14644.

[3] “LAMMPS - a flexible simulation tool for particle-based materials modelling at the atomic, meso, and continuum scales - ScienceDirect.” Accessed: Aug. 28, 2023. [Online]. Available: <https://www.sciencedirect.com/science/article/pii/S0010465521002836>

[4] G. Van Rossum and F. L. Drake Jr, *Python reference manual*. Centrum voor Wiskunde en Informatica Amsterdam, 1995.

[5] U of Edinburgh, “Edinburgh Compute and Data Facility web site.” [Online]. Available: [www.ecdf.ed.ac.uk](http://www.ecdf.ed.ac.uk)

[6] H. Zhang, M. Zhao, and Y. S. Lin, *Microporous Mesoporous Mater.*, **2019**, *279*, 201.

[7] H. Zhang, M. Zhao, Y. Yang, and Y. S. Lin, *Microporous Mesoporous Mater.*, **2019**, *288*, 109568.
